# Cardiac Hypertrophy

**Published:** 1891-01

**Authors:** 

**Affiliations:** Philla., Pa.


### CARDIAC HYPERTROPHY.

BY PROF. DACOSTA, PHILLA., PA.

Prof. DaCosta directs the diet to consist of milk, fish, vegetables. No coffee or tobacco. And

℞ Tinct. aconiti, gtt. j.
Tinct. verat veridis, gtt. iij.
Syrup zingiberis, gtt, vij. M.

Sis. This dose t. d.

### COUNTER INDICATIONS OF ANTIPYRINE.

Antipyrine is counter-indicated:

1. In all cases of feeble heart.

2. In diphtheria, when we have indications of the existence of myocarditis.

3. After profuse hemorrhage.

5. During menstruation, and in dysmenorrhœa.

5. In broncho-pneumonia in general, and in fibrous pneumonia complicated by pulmonary œdema.

6. In the last stages of pulmonary phthisis.

7. In all cases of extreme feebleness and emaciation, and in the advanced stages of chromic fevers.—*L' Union Medicale.*

### FOR TONSILITIS.

Dr. Hudson, of Stockton, Cal., in The Medical Record reports unfailing cures of tonsillitis in from eight to twelve hours with the following:

| | |
|---|---|
| Norwood's tincture of veratrum viride . . | 30 drops. |
| Sulphate of morphine . . . | 1 1-2 grain. |
| Distilled water . . . . . | 6 drachms. |

Of this, one teaspoonful is to be taken every hour for two hours, and then every two or three hours, as needed.

This is five drops of veratrum, and a quarter grain of morphine in a teaspoonful of water as the dose.—*Med. World.*

### ANTISEPTIC TREATMENT OF TONSILITIS.

℞ Borate of benzoate of soda, ʒ ijss.

Hot water, ℥ vij.

Dissolve and add:

Tincture of myrrh, gr. lxxv.
Blackberry syrup, ℥ j. M.
Ft. gargle.

Or the following:

℞ Resorcin, gr. xv.
Distilled water, f ℥ vij.
Blackberry syrup, f· ℥ j. M.
Ft. gargle.

Then brush over the tonsils, several time a day, with the following.

℞ Glycerine, ʒ v.

Camphor, gr. xv.

Carbolic acid, gr. xv. M.

Sig. Use as above, with camel's hair brush.

